# Underfeeding Alters Brain Tissue Synthesis Rate in a Rat Brain Injury Model

**DOI:** 10.3390/ijms241713195

**Published:** 2023-08-25

**Authors:** Casey C. Curl, Robert G. Leija, Jose A. Arevalo, Adam D. Osmond, Justin J. Duong, Daniela Kaufer, Michael A. Horning, George A. Brooks

**Affiliations:** Department of Integrative Biology, University of California at Berkeley, Berkeley, CA 94720-3140, USA; ccurl@berkeley.edu (C.C.C.); rgleija@berkeley.edu (R.G.L.); josearevalo@berkeley.edu (J.A.A.); adosmond@berkeley.edu (A.D.O.); danielak@berkeley.edu (D.K.);

**Keywords:** brain injury, standard of care, gluconeogenesis, recovery

## Abstract

Brain injuries (BI) are highly disruptive, often having long lasting effects. Inadequate standard of care (SOC) energy support in the hospital leads to dietary energy deficiencies in BI patients. However, it is unclear how underfeeding (UF) affects protein synthesis post-BI. Therefore, in a rat model, we addressed the issue of UF on the protein fractional synthesis rate (fSR) post-BI. Compared to ad libitum (AL)-fed animals, we found that UF decreased protein synthesis in hind-limb skeletal muscle and cortical mitochondrial and structural proteins (*p* ≤ 0.05). BI significantly increased protein synthesis in the left and right cortices (*p* ≤ 0.05), but suppressed protein synthesis in the cerebellum (*p* ≤ 0.05) as compared to non-injured sham animals. Compared to underfeeding alone, UF in conjunction with BI (UF+BI) caused increased protein synthesis rates in mitochondrial, cytosolic, and whole-tissue proteins of the cortical brain regions. The increased rates of protein synthesis found in the UF+BI group were mitigated by AL feeding, demonstrating that caloric adequacy alleviates the effects of BI on protein dynamics in cortical and cerebellar brain regions. This research provides evidence that underfeeding has a negative impact on brain healing post-BI and that protein reserves in uninjured tissues are mobilized to support cortical tissue repair following BI.

## 1. Introduction

In the United States alone, traumatic brain injuries accounted for 60,565 deaths in 2018; among those were 6688 adolescents between the ages of 15–17 [1]. Brain injuries (BI) often provoke long-lasting detrimental effects, and thereby, a poor quality of life for survivors [2,3,4,5]. During the acute phase of recovery from BI, nutritional status is frequently of low priority; consequently, patients can experience both underfeeding [6,7] and injury-induced metabolic crises [8,9,10,11,12,13,14,15,16,17,18,19]. With the potential poor outcomes associated with BI post-injury, it is important to understand the underlying metabolic effects and nutritional needs of BI patients.

Given the frequency of underfeeding following BI, it is surprising that little research has examined the brain protein synthesis rate post-injury. Utilizing short-term primed-continuous infusions of stable isotopically labeled amino acids [20,21], investigators have shown that brain protein synthesis decreased post-ischemia, a common consequence of BI [22,23]. Particularly, it has been observed that higher brain regions, such as the neocortex and hippocampus, had significantly depressed protein synthesis post-ischemia, while alternations in protein synthesis in the lower brain regions were minimal [21]. Thus, it appears that protein synthesis in the brain is heterogeneous and differs by region, mode, and location of injury.

With regard to energy substrate partitioning, post-BI, patients experience a hypermetabolic state characterized by elevated glucose and lactate concentrations [12,24,25]. Furthermore, when the impact force from a BI leads to the rupturing of the blood–brain barrier (BBB), there occurs an immense inflammatory response, subsequently leading to decreased synaptic plasticity and increased neuronal excitability [16,17,18]. As a result of the hypermetabolic state and the potential rupturing of the BBB post injury, it is unclear how underfeeding, typical during post-BI treatment [6,7], affects brain protein synthesis. Thus, using an animal model, we evaluated the protein synthesis rate to understand basic metabolic processes to improve standard of care (SOC) nutritional support following BI.

Chronic underfeeding leads to a negative nitrogen balance that is typically compensated for via skeletal muscle protein degradation [13,14,26,27]. Oxfeldt et al. demonstrated that 10 days of low energy feeding induced a negative nitrogen balance and decreased myofibrillar and sarcoplasmic skeletal muscle protein synthesis [28]. Additionally, after 7 days of caloric restriction, Yuan et al. observed reduced skeletal muscle and mitochondrial protein synthesis in Wistar rats [29]. Negative nitrogen balance may adversely affect brain and whole-body protein synthesis in underfed patients recovering from a BI.

The purpose of our study was to measure tissue and mitochondrial protein synthesis in different regions of the brain after BI in ad libitum (AL)-fed and underfed (UF) young animals. We hypothesized that (1) compared to sham animals, those that received BI would exhibit higher protein synthesis in all regions of the brain. (2) UF would suppress whole-body and brain protein synthesis. (3) UF+BI animals would have greater protein synthesis over UF controls, and inversely suppressed protein synthesis compared to AL BI animals.

## 2. Results

### 2.1. Behavioral Changes Due to BI

Effects of injury on animal behavior were published previously [19], but, a brief summary is presented here for reader’s convince. To test the severity of injury, we performed multiple state-of-the-art BI behavioral assessments. Of the numerous behavioral tests we performed, only a significant change in light sensitivity due to injury (*p* ≤ 0.05) was found. There were no changes in memory, balance, or grip strength due to injury (*p* ≥ 0.05).

### 2.2. Underfeeding Causes a Suppression of Protein Synthesis in Specific Tissue Regions

As expected, underfeeding suppressed protein synthesis rates in the gastrocnemius and the left cortex (Table 1). Specifically, fSR in the gastrocnemius was suppressed in both whole-tissue and mitochondrial fractions (*p* ≤ 0.05). Similarly, fSR in the left cortex was also depressed in whole-tissue and mitochondrial fractions (*p* ≤ 0.05). Other tissues studied (heart, right cortex, left and right cerebellum, and the hippocampus tissue), did not result in a suppressed fSR response to underfeeding. 

### 2.3. BI Causes an Increase in Protein Synthesis in the Cortical Regions of the Brain

Novel to our study, compared to sham injured animals, we found a significant increase in protein synthesis rates post-BI in the cortical regions of the brain (Table 2). BI increased protein fSR in all regions of the left cortex; whole (*p* ≤ 0.05), mitochondrial (*p* ≤ 0.05), and the cytosolic fraction (*p* ≤ 0.05) compared to sham injured animals. Similarly, in the right cortex, BI increased fSR in the whole-tissue (*p* ≤ 0.05) and the mitochondrial fraction (*p* ≤ 0.05). On the contrary, in the right cerebellum, BI decreased protein synthesis rates in the mitochondrial (*p* ≤ 0.05), cytosolic (*p* ≤ 0.05) fractions, and a trend in whole-tissue fraction (*p* ≤ 0.08) as compared to sham injured animals. 

### 2.4. Adequate Nutrition Normalizes Protein Synthesis Post-Injury

**Figure 1:**
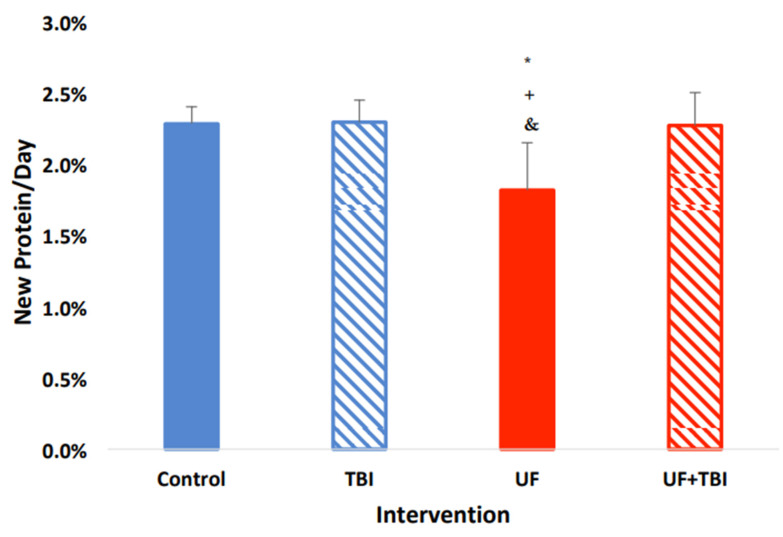
Combination of BI and UF Lead to an Increase in whole cortical protein synthesis rate. Left cortex protein fractional synthesis rate (%/day) in Control (AL + Sham Injury) as compared to fSR/day in intervention groups; Control (*n* = 3, solid blue), BI (*n* = 6, blue stripe), UF (*n* = 6, solid red), UF+BI (*n* = 5, red stripe). * Significantly different from Control (*p* ≤ 0.05), ^+^ significantly different from UF+BI (*p* ≤ 0.05), ^&^ significantly different from BI (*p* ≤ 0.05).

**Figure 2:**
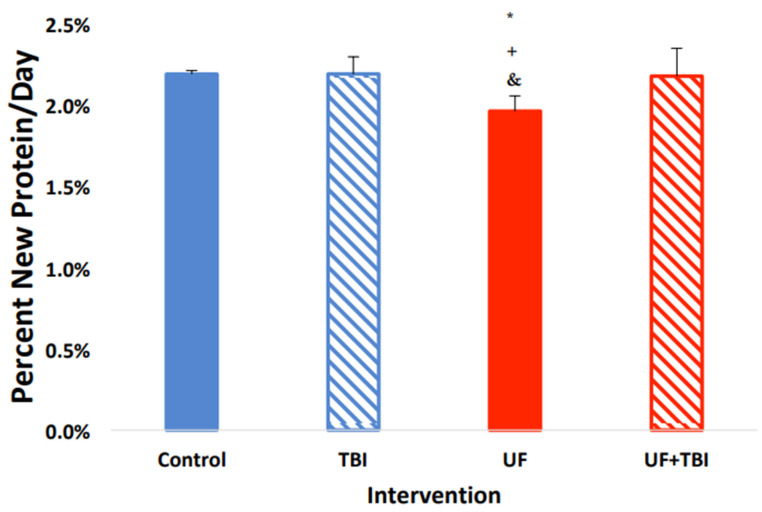
Combination of BI and UF Lead to an Increase in cortical mitochondrial protein synthesis Rate. Left cortex mitochondrial protein fractional syntheses rate (%/day) in Control (AL + Sham Injury) as compared to fSR/day in intervention groups; Control (*n* = 3, solid blue), BI (*n* = 6, blue stripe), UF (*n* = 6, solid red), UF+BI (*n* = 5, red stripe). * Significantly different from Control (*p* ≤ 0.05), ^+^ significantly different from UF+BI (*p* ≤ 0.05), ^&^ significantly different from BI (*p* ≤ 0.05).

**Figure 3:**
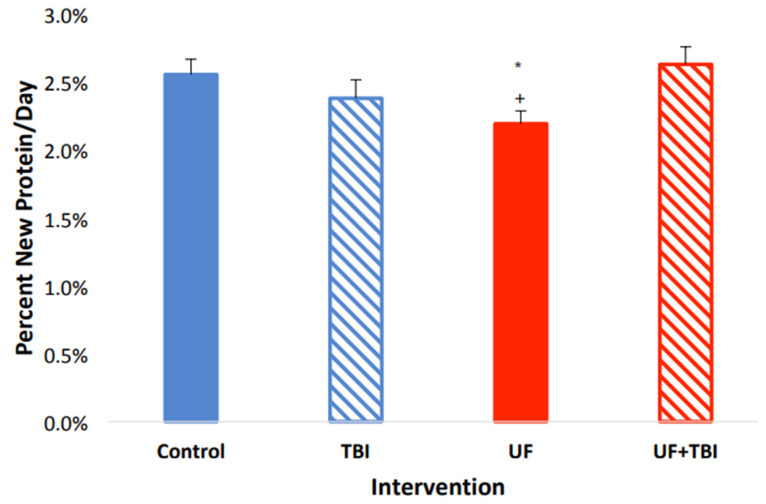
Combination of BI and UF leads to an increase in cortical cytosolic protein synthesis rate. Left cortex cytosolic protein fractional synthesis rate (%/day) in Control (AL + Sham Injury) as compared to fSR/day in intervention groups; Control (*n* = 3, solid blue), BI (*n* = 6, blue stripe), UF (*n* = 6, solid red), UF+BI (*n* = 5, red stripe). * Significantly different from Control (*p* ≤ 0.05), ^+^ significantly different from UF+BI (*p* ≤ 0.05).

With the significant suppression of protein synthesis seen in the UF group, and the upregulation of protein synthesis due to BI in the cortical regions of the brain, we decided to investigate the effects of both BI and feeding status on protein synthesis. We found a significant interaction between BI and feeding status on protein fSR within multiple tissues. Specifically, there was an interaction between BI and feeding in the whole (F = 6.86 *p* ≤ 0.05), and cytosolic (F = 30.46 *p* ≤ 0.001) fractions of the left cortex with trends in the mitochondrial fraction (F = 4.22 *p* ≤ 0.06). Similarly, we found there was an interaction between BI and feeding status in the whole right cortex fraction (F = 10.87 *p* ≤ 0.005) and the mitochondrial fraction in the left cerebellum (F = 10.143 *p* ≤ 0.01). To follow up on these interactions, we used pairwise comparisons in conjunction with a Tukey’s post hoc test to find group differences. Interestingly, we found that caloric adequacy, as indicated via AL feeding, normalized protein fSR post-BI in all tissues compared to ad libitum-fed control animals (all fSR *p* > 0.05, Table 3). In contrast, nutritional inadequacy, as indicated via UF, caused a hypermetabolic response in multiple regions of the brain post-BI compared to the UF sham injury group. Specifically, we saw an increase in protein fSR in all fractions of the left cortex: whole (*p* ≤ 0.05), mitochondrial (*p* ≤ 0.05), and cytosolic (*p* ≤ 0.001) fractions (Figure 1, Figure 2 and Figure 3). We also observed an increased protein fSR in the whole-tissue and mitochondrial fractions of the right cortex (*p* ≤ 0.05). Lastly, we detected a significant increase in mitochondrial protein fSR in the left cerebellum (*p* ≤ 0.05). Interestingly, protein fSR in the UF+BI group was elevated to the point that it was indistinguishable from ad libitum-fed sham injured control animals in any of the tissue/fractions (all fSR *p* > 0.05). With the significant increases in protein synthesis in the UF+BI group, we can infer there is increased metabolic activity in this group to compensate for the consequences of the injury, causing an increased protein fSR compared to the basal rate seen with underfeeding. 

### 2.5. The Relationship between Underfeeding and fSR, Evidence That UF+BI Leads to an Altered Protein Partitioning

To further demonstrate the effect UF had on protein synthesis post-BI, we correlated fSR with an absolute value of UF, as measured by fractional gluconeogenesis (fGNG) [19]. We found there was a significant negative correlation between fSR and fGNG in both the whole and mitochondrial fraction of the gastrocnemius muscle (*p* ≤ 0.05, Table 4). Also, there was a trend for a negative correlation between fSR and fGNG in the left cortex whole and mitochondrial fractions (*p* ≤ 0.07), indicating caloric inadequacy decreases skeletal muscle fSR. Yet, because there were significant differences in fSR found between UF+BI and UF sham injured animals, as seen in the data above (Section 2.4), we decided to run correlations between fSR and fGNG, omitting the UF+BI group to understand how much UF effected fSR post-BI. Consequently, we found the correlations became stronger between skeletal muscle fSR and fGNG (*p* ≤ 0.01). Furthermore, we found a significant negative correlation between fSR and fGNG in both the left cortex and the right cortex whole and mitochondrial fractions (*p* ≤ 0.01), with a trend towards a negative correlation between fSR and fGNG in the whole-tissue fraction of the left cerebellum (*p* ≤ 0.06). The difference in the correlations, as shown in Table 4, is evidence that protein partitioning may be altered when UF is administered following a BI.

## 3. Discussion

The purpose of this study was to assess the role underfeeding had on protein synthesis in young rats recovering from a BI. Our efforts corroborated results of those who observed that acute UF depressed whole-tissue and mitochondrial protein synthesis [28,29]. In addition, we showed that cortical protein synthesis was elevated post-BI, while the protein synthesis in the right cerebellum was downregulated post-injury. Interestingly, we found that protein synthesis was elevated in the cortical regions of the brain post-BI when UF was imposed. Not surprisingly, however, those effects were mitigated by caloric adequacy as established by ad libitum (AL) feeding. Given our results, it appeared that protein pools in non-injured body tissues were sacrificed to provide essential macronutrients and energy to protect the brain during post-BI recovery. Additionally, inadequate treatment results in an energy deficit, as seen when SOC feeding protocols are not adhered to or are insufficient. 

Our study used a rodent model designed to mimic patient care in a state-of-the-art Neurological Intensive Care Unit (Neural ICU) in which underfeeding contributed to the post-injury metabolic crisis. Specifically, Glenn and colleagues showed how the underfeeding of BI patients caused a significant increase in reliance of endogenous production of glucose, via gluconeogenesis from lactate to maintain blood glucose levels [15]. Building off the findings from Glenn and colleagues, we observed a significant increase in fGNG with underfeeding in BI animals as compared to AL-fed BI animals [19]. While UF and UF+BI animals displayed similar increases in fGNG with underfeeding [19], UF+BI animals had increased cortical protein synthesis over UF animals, indicating greater metabolic requirements for protein synthesis. Yet, Control and AL-fed BI animals showed no difference in protein synthesis, indicating that metabolic requirements were met during recovery. Thus, it seems there was a detrimental effect of underfeeding on animal recovery post-injury. 

### 3.1. Clinical Ramifications of Acute Underfeeding Post-Injury

Unfortunately, underfeeding is common post-BI [6,7,13,14], a situation that leads to higher gluconeogenic rates [9] and negative nitrogen balance [13,14]. These catabolic events result in increased protein catabolism and mobilization of endogenous nutrient stores to compensate for the lack of caloric supplementation. Regardless of diet composition, if energy expenditure exceeds caloric intake, patients enter a state of negative nitrogen balance [26,30]. In our study, UF animals exhibited decreased body weights and increased fGNG [19], indicative of negative nitrogen balance. Additionally, UF appeared to suppress skeletal muscle protein synthesis, possibly due to resource diversion for the brain, i.e., the corpus feeds the brain.

### 3.2. Changes in Brain Region Protein Dynamics Post-BI

The cortical region of the brain is responsible for executive functions and coordinating information with the sub-cortical regions [31]. Encephalization of the brain resulted in an increased prefrontal cortex and cortical region size and increased neural connectivity and activity in mammals [32]. With the significant importance placed on the cortical section of the brain, our data suggest that the cortex is crucially important during post-BI recovery. Compared to sham animals, we showed increased protein synthesis in both the left and right cortex of BI animals, while simultaneously showing decreased protein synthesis in the right cerebellum. With the increased cortical protein synthesis, and decreased synthesis in the cerebellum, there may exist an altered substrate partition to preserve the executive function of the brain, thereby sacrificing other regions of the brain and other metabolically active tissues such as the skeletal muscle.

Along with significant increases in protein synthesis in the whole tissue of the cortex, there were also significant increases in protein synthesis in the mitochondrial and cytosolic fractions of the cortex. Specifically, with UF+BI, we saw an immense increase in mitochondrial and cytosolic protein synthesis as compared to the UF group. A possible reason for increased protein synthesis in these cellular compartments could be increased metabolic requirements post-BI. The brain’s energetic requirement is derived almost exclusively from glucose and lactate oxidation [33,34] that are increased post-BI in patients treated with SOC nutrition where the majority of energy (68%) comes from lactate, either directly via oxidation or indirectly via the conversion of lactate to glucose via gluconeogenesis [15]. 

Building off earlier work on the Lactate Shuttle hypothesis [35,36,37], Magistretti and colleagues demonstrated that astrocytes take up glucose from the blood and produced lactate that is shuttled into neurons for energy to support glutamatergic signaling. Together with lactate from the cerebral circulation, lactate represents the main exogenous fuel energy source for neurons. Accordingly, they proposed an Astrocyte-Neuron Lactate Shuttle (ANLS) [33,38,39]. Consistent with the idea of increased reliance on lactate as an energy source post-BI, Prinz et al. found an increase in cerebral lactate transporter (MCT 2) expression [40]. The elevated protein synthesis of the mitochondrial and cytosolic fractions may be needed to increase transporter expression, such as MCTs. Increased MCT expression would serve to shuttle lactate and other monocarboxylates such as pyruvate and ketones to support the metabolic requirements of cortical tissues. In our current study, we could not determine which proteins were up- or downregulated due to BI; future research utilizing D_2_O, and proteomics could tease out the effects of BI and UF at the level of the individual proteins such as MCTs.

### 3.3. Limitations

The study was designed to induce a moderate-to-severe injury to mimic a sports injury as encountered by young athletes. However, based on post-injury behavioral testing, it appeared that we administered a mild BI [19]. Still, despite the minimal post-injury behavioral changes detected, we found significant changes in protein synthesis due to BI, especially in the cortical region of the UF+BI group compared to the cortical region of UF sham injured animals. It is possible that we did not see similar results in the *ad libitum*-fed BI group because the rats may have been fully healed 13 days after injury. We also may not have been able to detect the changes in protein synthesis rates between UF+BI animals and AL-fed animals because of the young rat ages. We measured fSR during the rat’s heightened growth period; results might have been different in older animals that had achieved weight stability. Lastly, while we only had three control animals, our protein turnover rates are similar to those of the only other study that has measured whole brain fSR [41]. Thus, we believe our results are valid. 

Regarding our utilization of D_2_O as a measurement for protein turnover, the method assumes a steady state protein turnover where the protein rate of appearance equals the protein rate of disappearance [42]. Although there were changes to the animal’s bodyweight, there were no significant differences in the weights of the gastrocnemius and heart muscles excised from any group, experimental or control group. Thus, we believe that the steady-state assumption to use fSR as a parameter of protein synthesis within groups (i.e., AL, or UF) was justified. 

## 4. Materials and Methods

### 4.1. Overall Study Design

The overall design of the study was published in Curl et al. [19], but is presented here for the convenience of the reader. Procedures on animals were approved by the University of California, Berkeley Animal Care and Use Committee (2018-08-11312). Male Sprague Dawley rats were purchased from Charles River (Wilmington, MA, USA) at 49 days of age and housed individually. Cages were maintained at a constant temperature and humidity with a 12 h light–dark cycle (Light: 7:00 a.m. to 7:00 p.m.). Animals were given free access to 6% deuterium oxide-labeled drinking water (D_2_O) for the entirety of the study (day 1–22), and AL access to standard chow mix (PicoLab Rodent Diet 20: 62:13:25% carbohydrate, fat, and protein) for the first 7 days. On day 8, animals were randomly assigned to 4 groups based on injury and nutritional interventions: 1. Sham injury control with AL feeding (Control *n* = 3), 2. BI and AL feeding (BI *n* = 6), 3. BI and half-ration underfeeding (UF+BI *n* = 5), and 4. Sham injury and underfeeding (UF *n* = 6). After group randomization, a free rotation closed-head BI was induced [43]. Following BI, individual rat weights were recorded periodically throughout the study (Table 5). The half-ration, UF treatment was designed to mimic human patient treatment in a Neuro ICU [9,25]. For the first 7 days, food trays were weighed to calculate the half ration for the groups that received underfeeding. Then, the average of each rat’s daily food consumption prior to BI was calculated and rounded up to the nearest half gram and divided by two to determine the UF group half-ration daily allotment.

D_2_O remained continuously available for the animals, and food was removed from animal cages 24 h before euthanasia. Animals were euthanized on day 22 of the experiment at the age of 71 days old via carbon dioxide asphyxiation followed by decapitation. Subsequently, blood was collected in EDTA tubes and spun at 3000× *g* for 18 min to separate the plasma which was stored at −20 °C until analysis. All tissues (gastrocnemius, heart, cortex, hippocampus, and cerebellum) were collected immediately post-euthanasia and flash frozen with liquid nitrogen and stored at −80 °C until analyzed.

### 4.2. Brain Injury Model

We utilized a modified free rotation, closed-head brain injury method to induce injury [43]. Prior to impact, the animals were anesthetized using 3.5% isoflurane atomized in oxygen at a low rate of 1 L/min for approximately 15 min. If the breathing rate remained elevated or a toe-pinch reflex was present, the animals continued under anesthesia for an additional minute or until the toe-pinch reflex was no longer detected. Animals were then quickly moved to a perforated foil platform 8 cm above a 7.6 cm thick medium-density foam pad in a prone position. The bolt was positioned on the rat’s head along the midline and aligned with the ears to target between lambda and bregma skull landmarks. After confirming the toe-pinch reflex had not been regained, a 450 g weight was dropped from 135 cm onto a 3 cm of bolt throw. Sham animals underwent the same course of anesthesia and placement on the apparatus with no weight drop. Immediately post impact, animals were returned to a clean cage in the supine position and observed.

### 4.3. Labeled Water and Body Water Enrichment Analysis

The utilization of D_2_O allows the assessment of protein turnover in multiple tissues and multiple fractions within each tissue. The assessment of body water enrichment was described by Miller et al. [42,44]. Briefly, 120 μL of plasma was placed into the cap of inverted screw-capped tubes and placed in a heat block for overnight distillation at 80 °C. Distilled samples were diluted 1:300 in doubly distilled (dd) H_2_O and analyzed on a liquid water isotope analyzer (Los Gatos Research, Los Gatos, CA, USA) against a standard curve prepared with samples containing different percentages of D_2_O. 

### 4.4. Tissue Isolation

Tissues from the gastrocnemius heart, hippocampus, left cortex, right cortex, and the left cerebellum and right cerebellum were fractioned utilizing differential centrifugation as previously published [45]. Tissues were homogenized in a 1:10 isolation buffer (250 mM mannitol, 10 mM EDTA, 45 mM Tris-HCL, and 5 mM tris base; pH to 7.4) with phosphatase and protease inhibitors (ThermoFisher, Waltham, MA, USA) using a pestle homogenizer. An aliquot of the whole-tissue homogenate was placed in a collection vial and stored at −80 °C until further analysis. The remaining tissue homogenates were then centrifuged at low speed (800× *g*) for 10 min at 4 °C. The supernatant was extracted and spun at a high speed (10,000× *g*) to isolate the mitochondrial pellet while the pellet from the low-speed spin was discarded. The supernatant from the high-speed spin, containing the cytosol and plasma membrane fragments, was saved and kept at −80 °C until analysis. The mitochondrial pellet was resuspended in 100 μL of isolation buffer and then frozen at −80 °C until analysis.

### 4.5. Measuring Protein Fractional Synthesis Rate

Tissue fractions were hydrolyzed in 6 N HCL at 120 °C overnight. The hydrolysates were dried and then suspended in 1 mL of 50% acetonitrile and 50 mM K_2_PO_4_ (pH to 11). Subsequently, 20 μL of pentafluorobenzyl bromide was added to the sample. The sample was sealed, vortexed and heated at 100 °C for 1 h to derivatize. After an hour, the sample was cooled at room temperature followed by the addition of 500 μL of ethyl acetate. Consequently, the organic layer was extracted and dried under N_2_ gas. The dried organic layer was then resuspended in 50 to 100 μL of ethyl acetate and was made ready for analysis [46].

Isotopic enrichments of alanine were measured on an Agilent 6890/5973 Gas Chromatography-Mass-Spectrometry (GC-MS) utilizing a DB 225 chromatography column and helium carrier gas. Methane was used for negative chemical ionization (NCI) and selected ion monitoring (SIM) was used to detect masses 448, 449, and 450. The starting oven temperature was 100 °C, increasing 30 °C/min to 220 °C and held for 13.5 min. The mass to charge ratios (*m*/*z*) of 448, 449, and 450 were monitored for the pentafluorobenzyl-N, N-di(pentafluorobenzyl) alanine derivative.

Newly synthesized protein fraction (f) was calculated from the enrichment of the alanine derivatives bound to muscle proteins over the labeling period. The enriched fractions (f) were then divided by the precursor enrichment (p), using body water enrichment in conjunction with MIDA [47].

### 4.6. Statistical Analyses

We used SPSS (IBM SPSS Statistics for Macintosh, Version 27.0, IBM Corp, Armonk, NY, USA) for all statistical analyses. To examine the differences in fSR between groups (i.e., AL vs. UF, or Sham vs. BI), we utilized T-Tests and set significance to *p* ≤ 0.05. To assess significance of differences in fSR comparing both feeding and BI as our independent variables, we used an analysis of variance (ANOVA) and a Tukey’s post hoc analysis to maintain probability *p* ≤ 0.05 for multiple comparisons. Pearson correlation coefficients were used to determine the relationship between fSR and fGNG, a marker of caloric adequacy [19].

## 5. Conclusions

Brain injuries induce a hypermetabolic state [25] that is exacerbated by underfeeding. Paradoxically, fSR was elevated in cortices of underfed BI rats. However, adequate, ad libitum feeding allowed for the normalization of protein fractional syntheses rates in rat cortices post-injury. Results from this study should help inform practitioners on the need for adequate nutritional support post-BI. 

## Figures and Tables

**Table 1:** Underfeeding suppresses relative daily skeletal muscle and cortical protein synthesis rates.

Tissue Fraction	Ad Libitum-Fed Animals	Underfed Animals
Gastrocnemius		
Whole	2.70 ± 0.17%	2.38 ± 0.39% *
Mitochondria	2.80 ± 0.16%	2.56 ± 0.20 *
Cytosol	2.76 ± 0.38%	2.71 ± 0.19%
Heart		
Whole	2.40 ± 0.30%	2.34 ± 0.25%
Mitochondria	1.86 ± 0.66%	2.10 ± 0.57%
Cytosol	2.38 ± 0.33%	2.29 ± 0.24%
Left Cortex		
Whole	2.29 ± 0.14%	2.02 ± 0.36% *
Mitochondria	2.19 ± 0.08%	2.16 ± 0.17% *
Cytosol	2.43 ± 0.14%	2.38 ± 0.24%
Right Cortex		
Whole	2.19 ± 0.10%	2.10 ± 0.24%
Mitochondria	2.24 ± 0.10%	2.19 ± 0.19%
Cytosol	2.33 ± 0.29%	2.19 ± 0.33%
Left Cerebellum		
Whole	2.14 ± 0.14%	2.00 ± 0.29%
Mitochondria	2.29 ± 0.14%	2.29 ± 0.19%
Cytosol	2.48 ± 0.14%	2.43 ± 0.19%
Right Cerebellum		
Whole	1.81 ± 0.42%	1.71 ± 0.38%
Mitochondria	1.81 ± 0.57%	1.76 ± 0.52%
Cytosol	2.29 ± 0.43%	2.24 ± 0.43%
Hippocampus		
Whole	2.33 ± 0.14%	2.38 ± 0.10%
Mitochondria	2.33 ± 0.24%	2.38 ± 0.10%
Cytosol	2.67 ± 0.10%	2.67 ± 0.19%

Protein fSR/% day depending on feeding; Ad libitum-fed animals (*n* = 9: Control = 3, BI = 6) vs. underfed animals (*n* = 11: UF = 6, UF+BI = 5) in the gastrocnemius, heart, left and right cortex, left and right cerebellum, and the hippocampus tissue fractions (whole, mitochondrial, and cytoplasmic/cytosolic). * Significantly different than ad libitum-fed *p* ≤ 0.05.

**Table 2:** Brain injury significantly increases relative cortical fSR.

Tissue Fraction	Sham Animals	BI Animals
Gastrocnemius		
Whole	2.53 ± 0.29%	2.54 ± 0.39%
Mitochondria	2.64 ± 0.25%	2.68 ± 0.20%
Cytosol	2.60 ± 0.35%	2.85 ± 0.18%
Heart		
Whole	2.48 ± 0.30%	2.28 ± 0.22%
Mitochondria	2.24 ± 0.46%	1.76 ± 0.67%
Cytosol	2.43 ± 0.25%	2.25 ± 0.32%
Left Cortex		
Whole	1.93 ± 0.38%	2.28 ± 0.18% *
Mitochondria	2.04 ± 0.12%	2.19 ± 0.14% *
Cytosol	2.31 ± 0.20%	2.49 ± 0.18% *
Right Cortex		
Whole	2.05 ± 0.19%	2.23 ± 0.14% *
Mitochondria	2.12 ± 0.09%	2.28 ± 0.14% *
Cytosol	2.36 ± 0.18%	2.14 ± 0.37%
Left Cerebellum		
Whole	2.11 ± 0.12%	2.05 ± 0.25%
Mitochondria	2.21 ± 0.16%	2.33 ± 0.14%
Cytosol	2.41 ± 0.15%	2.48 ± 0.17%
Right Cerebellum		
Whole	1.92 ± 0.34%	1.61 ± 0.41% ^&^
Mitochondria	2.08 ± 0.32%	1.58 ± 0.57 *
Cytosol	2.49 ± 0.13%	2.05 ± 0.45% *
Hippocampus		
Whole	2.39 ± 0.14%	2.33 ± 0.10%
Mitochondria	2.37 ± 0.22%	2.33 ± 0.14%
Cytosol	2.65 ± 0.20%	2.68 ± 0.14%

Effects of injury model on protein fSR/% day; Sham injured (Sham Animals *n* = 9: AL fed = 3, UF = 6) vs. BI animals (*n* = 11: BI = 6, UF+BI = 5) in the gastrocnemius, heart, left and right cortex, left and right cerebellum, and the hippocampus tissue fractions (whole, mitochondrial, and cytoplasmic/cytosolic. * Significantly different than Sham Injury *p* ≤ 0.05, ^&^ trended to be different than Sham Injury *p* ≤ 0.10.

**Table 3:** The combination of BI and UF alter cortical relative fSR.

Tissue Fraction	Control	BI	UF	BI+UF
Gastrocnemius				
Whole	2.76 ± 0.32%	2.66 ± 0.05%	2.37 ± 0.18%	2.39 ± 0.57%
Mitochondria	2.91 ± 0.22%	2.75 ± 0.11%	2.51 ± 0.14% *	2.61 ± 0.27%
Cytosol	2.50 ± 0.63%	2.90 ± 0.18%	2.64 ± 0.17%	2.78 ± 0.18%
Heart				
Whole	2.63 ± 0.42%	2.29 ± 0.16%	2.40 ± 0.22%	2.27 ± 0.30%
Mitochondria	2.14 ± 0.82%	1.70 ± 0.64%	2.29 ± 0.23%	1.84± 0.77%
Cytosol	2.66 ± 0.25%	2.22 ± 0.31%	2.31 ± 0.15%	2.27 ± 0.37%
Left Cortex				
Whole	2.28 ± 0.12%	2.29 ± 0.15%	1.81 ± 0.33% *^+&^	2.28 ± 0.23%
Mitochondria	2.17% ± 0.02%	2.19 ± 0.10%	1.96 ± 0.10% *^+&^	2.17± 0.17%
Cytosol	2.55 ± 0.11%	2.38 ± 0.13%	2.19 ± 0.09% *^+^	2.63 ± 0.13%
Right Cortex				
Whole	2.24 ± 0.06%	2.167 ± 0.11%	1.96 ± 0.14% *^+&^	2.29 ± 0.17%
Mitochondria	2.20 ± 0.07%	2.25 ± 0.13%	2.07 ± 0.21% ^+^	2.29 ± 0.21%
Cytosol	2.56 ± 0.05%	2.21 ± 0.28%	2.26 ± 0.12%	2.06 ± 0.48%
Left Cerebellum				
Whole	2.23 ± 0.20%	2.08 ± 0.10%	1.86 ± 0.35% ^+^	2.14 ± 0.16%
Mitochondria	2.35 ± 0.23%	2.26 ± 0.07%	2.14 ± 0. 06%	2.42 ±0.15%
Cytosol	2.51 ± 0.15%	2.51 ± 0.15%	2.51 ± 0.15%	2.51 ± 0.15%
Right Cerebellum				
Whole	2.44 ± 0.13%	2.44 ± 0.13%	2.44 ± 0.13%	2.44 ± 0.13%
Mitochondria	2.36 ± 0.13%	2.36 ± 0.13%	2.36 ± 0.13%	2.36 ± 0.13%
Cytosol	2.54 ± 0.22%	2.54 ± 0.22%	2.54 ± 0.22%	2.54 ± 0.22%
Hippocampus				
Whole	2.47 ± 0.17%	2.28 ± 0.07%	2.35 ± 0.11%	2.39 ±0.11%
Mitochondria	2.32 ± 0.41%	2.31 ± 0.16%	2.39 ± 0.07%	2.35 ± 0.19%
Cytosol	2.73 ± 0.09%	2.60 ± 0.10%	2.60 ± 0.23%	2.76 ± 0.15%

The combined effects of feeding status and injury model on protein fSR/% day in the gastrocnemius, heart, left and right cortex, left and right cerebellum and the hippocampus tissue fractions (whole, mitochondrial and cytoplasmic/cytosolic); Control (*n* = 3), BI (*n* = 6), UF (*n* = 6), UF+BI (*n* = 5). * Significantly different from Control (*p* ≤ 0.05), ^+^ significantly different from UF+BI (*p* ≤ 0.05), ^&^ significantly different from BI (*p* ≤ 0.05).

**Table 4:** Increased UF+BI fSR decreased fSR and fGNG correlations.

Tissue	With UF+BI	Without UF+BI	Difference
Gastrocnemius			
Whole	−0.51 *	−0.63 **	0.12
Mitochondria	−0.50 *	−0.59 **	0.08
Cytosol	−0.23	−0.28	0.05
Heart			
Whole	0.04	0.18	0.14
Mitochondria	0.31	0.50 ^+^	0.19
Cytosol	−0.02	−0.01	0.01
Left Cortex			
Whole	−0.41 ^+^	−0.63 **	0.22
Mitochondria	−0.47 *	−0.79 **	0.32
Cytosol	−0.13	−0.49 ^+^	0.35
Right Cortex			
Whole	−0.28	−0.61 **	0.33
Mitochondria	−0.25	−0.66 **	0.4
Cytosol	−0.18	0.06	0.25
Left Cerebellum			
Whole	−0.31	−0.49 ^+^	0.18
Mitochondria	−0.03	−0.31	0.28
Cytosol	0	−0.14	0.14
Right Cerebellum			
Whole	0.09	0.15	0.07
Mitochondria	0.12	0.38	0.27
Cytosol	0.02	0.21	0.19
Hippocampus			
Whole	0.33	0.26	0.07
Mitochondria	0.37	0.43	0.06
Cytosol	0.08	0.01	0.07

Correlation between fSR and fGNG with UF+BI group included (*n* = 20) vs. UF+BI omitted (*n* = 15) in the gastrocnemius, heart, left and right cortex, left and right cerebellum and the hippocampus tissue fractions (whole, mitochondrial and cytoplasmic/cytosolic). * Significant correlation *p* ≤ 0.05, ** significant correlation *p* ≤ 0.01, ^+^ trended to be significant *p* ≤ 0.06.

**Table 5:** Underfeeding decreases body weight.

Group	Number of Animals	Pre-Intervention	24 h Post-Intervention	13 Days Post-Intervention	Total Weight Change
Control	3	249.3 ± 2.1 g	248.7 ± 0.6 g	294.3 ± 6.7 g	45.0 ± 6.2 g
BI	6	252.2 ± 15.8 g	241.2 ± 15.5 g *	285.3 ± 16.1 g	33.2 ± 20.6 g
UF	6	292.16 ± 14.8 g *^+^^	291.5 ± 15.3 g	278.3 ± 10.6 g	−13.83 ± 13.2 g *^+^
UF+BI	5	244.8 ± 13.9 g	233.0 ± 12.1 g *	216.6015.8 g *^$+^	−28.20 ± 7.0 g *^+^

Bodyweight changes (grams) from pre-intervention to 24-h, 13-day post intervention and total bodyweight changes. * Significantly different compared to control *p* ≤ 0.05, ^+^ significantly different compared to BI *p* ≤ 0.05, ^$^ significantly different compared to UF *p* ≤ 0.05, ^^^ significantly different compared to UF+BI *p* ≤ 0.05.
